# Comparison of positive SARS-CoV-2 incidence rate with environmental and socioeconomic factors in northern Illinois^☆^

**DOI:** 10.1016/j.heliyon.2021.e07806

**Published:** 2021-08-14

**Authors:** Martha Menchaca, Frank Pagone, Serap Erdal

**Affiliations:** a School of Medicine, University of Illinois at Chicago, 1740 West Taylor, M/C 931, Chicago, Il 60612, USA; b RHP Risk Management Inc., 8745 W, Higgins Rd. Suite 320, Chicago, IL 60631, USA; c Environmental and Occupational Health Sciences, School of Public Health, University of Illinois at Chicago, 1603 West Taylor Street, M/C 923 Chicago, IL 60612, USA

**Keywords:** Environmental pollution, PM_2.5_, SARS-CoV-2, Health, Socioeconomic

## Abstract

Early studies showed positive associations fine particulate matter (PM_2.5),_ course particulate matter PM_10,_ nitrogen dioxide (NO_2_) and Ozone (O_3_) concentrations with severe acute respiratory syndrome coronavirus 2 (SARS-CoV-2) confirmed cases in the United States. One study showed that a1 μg/m3 increase in PM_2.5_ is associated with an 8% increase in the COVID-19 death rate. Specifically, Chicago and surrounding suburbs have been labeled hot spots in the United States and correlation with air pollutants concentration will help identify specific communities most at risk. A number of studies have identified demographic variables associated with increased positive SARS-CoV-2 and the importance of air quality and socioeconomic factors must be further understood for more targeted public health responses. The results of this analysis noted positive relationships between zip code SARS-CoV-2 incidence rate and environmental and demographic EJ indicators. Evaluation of race and SARS-CoV-2 incidence rate at the zip code level found positive moderate correlations for ethnic minority individuals.

## Highlights

• Positive relationships between SARS-CoV-2 incidence rate and environmental indicators.
• Positive correlation between SARS-CoV-2 incidence rate and race and ethnic indicators.
• Resources can be efficiently coordinated to areas with highest risk based factors.

## 1 Introduction

Since the world-wide pandemic of the severe acute respiratory syndrome coronavirus 2 (SARS-CoV-2) novel leading to coronavirus pneumonia (COVID-19) began in December 2019, it has spread around the globe and overburdened the worldwide health systems and the World Health Organization (WHO) declared a global pandemic in March 2020. A study using an ecological regression analysis found that counties with higher PM 2.5 exposure were positively associated with higher COVID- 19 mortality rates (Wu et al., 2020a, b, c).

As researchers continue to investigate aspects of the disease and various factors influencing health outcomes, environmental pollution research studies have determined that many of the pre-existing health conditions that may increase the risk of death in individuals with COVID-19.

It has been well documented in the scientific literature that long term exposure to air pollutants (e.g., nitrogen dioxide (NO_2,_ sulfur dioxide (SO_2_) and fine particulate matter (PM_2.5_) has adverse cardiovascular and respiratory health effects and increases mortality risk (Hu et al., 2015; Xing et al., 2016). Additionally, asthma and obstructive lung disease are exacerbated by affected by long-term exposure to air pollution. While recent systematic reviews and meta-analysis of comorbidities have found that asthma and obstructive lung disease are not associated with higher COVID-10 severity or worse prognosis, patients with cardiovascular disease are at higher risk of mortality from COVID19 (Liu et al., 2020; SSentongo et al., 2020).

A number of studies conducted to date reported evidence postulating higher particulate matter concentrations is a risk factor for severe acute respiratory syndrome coronavirus 2 (SARS-CoV-2) novel leading to coronavirus pneumonia COVID-19 incidence and increased mortality rates. More specifically, research explored associations between measured daily ambient air pollutant concentrations in 120 cities in China and daily COVID-19 confirmed cases from January 23, 2020 to February 29, 2020 using a generalized additive model (Zhu et al., 2020). Additionally, using a multivariable negative binomial regression model, researchers found an association between nitrogen dioxide and COVID-19 mortality in northern Italy (Filippini et al., 2020). In another study using spatio-temporal models to identify the influence of biodiversity, temperature, and precipitation and fitted generalized linear mixed models to identify the effects of environmental variables found that there is a relationship of loss of biodiversity, high level of air pollutants, and diminished air quality with COVID-19 infection spread and mortality (Fernandez et al., 2021).

A national study evaluating the U.S. estimated relationship between county-level 2000–2016 average PM _2.5_ concentrations estimated using satellite, modeled, and monitored data and COVID-19 mortality rates up to April 22, 2020 in 3000 U.S. counties (representing 98% of the population) used a negative binomial model found that small increase in long-term exposure to PM 2.5 leads to a large increase in the COVID-19 death rate after controlling for numerous confounding variables (e.g., population size, age distribution, population density, time since the beginning of the outbreak, time since state's issuance of stay-at-home order, hospital beds, number of individuals tested, weather, and socioeconomic and behavioral variables such as obesity and smoking (Wu et al., 2020a, b, c). This first U.S.-based study estimated that a 1 μg/m^3^ increase in long-term PM_2.5_ concentrations is associated with an 8% increase in the COVID-19 death rate, suggesting that long-term exposure to PM _2.5_ in air increases vulnerability to the most severe COVID-19 outcomes. In comparison, a study found that a 1 *μ*g/m^3^ increase in long-term PM_2.5_ concentrations was associated with a 0.73% increase in the rate of all-cause mortality for 60 million Americans older than 65 years of age (Di et al., 2017).

Similarly in an ecological study found that exposure to PM was significantly associated with the COVID-19 incidence and excess mortality during the first wave of the outbreak in Lombardy, Italy (De Angelis et al., 2021). A review of published literature evaluating relationship between some air pollutants, PM2.5, PM10 and NO2, and COVID-19 outbreak found that there was an association between PM2.5 and NO2 as triggering of the COVID-19 spread and lethality (Copat et al., 2020).

However, other recent work utilizing a binomial models to estimate the association between long term (2010–2016) county-level exposures to NO2, PM2.5, and O3 and county-level COVID-19 case fatality and mortality rates in the United States found that there was an association between NO2 and severity of COVID 19 outcomes but independent of long-term PM2.5 and O3 exposure (Liang et al., 2020).

The Northeastern Illinois metropolitan area with a population of over five million individuals, has been identified as a hot spot for COVID-19, with approximately 105,000 reported total cases and 4,800 deaths as of July 2020 (approximately 59% of all cases recorded for Illinois), and approximately 2,000 cases per 100,000 individuals, which is one of the highest COVID-19 incidence rates in the United States (Cook County Department of Public Health (CCDPH) 2020). Additionally, there is a diverse ethnic population and includes an array of industrial, commercial, and residential areas containing a variety of environmental pollution sources which include major highways, and manufacturing and industrial corridors. Air pollution emitted from transportation source include NO _2,_ particulate matter (PM), diesel particulate matter (DPM) and volatile organic compounds (VOCs) and both manufacturing and industrial sources can emit a variety of pollutants including NO_2_, sulfur dioxide (SO_2_ PM, VOCs, carbon monoxide (CO), and toxic air pollutants (e.g. ethylene oxide, chloropropene and other volatile and semi-volatile organic pollutants) (USEPA, 2020a, b). In addition, harmful ground level ozone is formed by a chemical reaction between nitrogen oxides (NO_x_) and VOCs which occurs when pollutants emitted by transportation, manufacturing, industrial and other sources chemically react in the atmosphere in the presence of sunlight (USEPA, 2020a, b). The combination of potential pollution sources, population diversity and elevatated COVID-19 incidence in Northeastern Illinois make this region an ideal laboratory for the evaluation of the relationship between COVID-19 incidence rate and environmental and demographic factors.

To evaluate the relationship between the COVID-19 incidence rates and both environmental and demographic factors within the Northeastern Counties in Illinois, the Illinois Department of Public Health (IDPH) (2020) COVID-19 Zip Code = level data of June 23, 2020 and the United States Environmental Protection Agency (USEPA) Environmental Justice Screening and Mapping Tool (EJSCREEN) data were spatially and statistically analyzed. The EJSCREEN database provides a nationally consistent dataset, which has been used as a data source for numerous environmental justice (EJ) research studies, that combines both environmental and demographic EJ indicators on a census block group level in the U.S (Ryder et al., 2020; Hu et al., 2020).

## 2 Materials and methods

To evaluate the spatial relationship between the COVID-19 incidence rates as reported by the State of Illinois Department of Health and environmental and demographic factors within Cook, DuPage, Lake, Will, Kane, and McHenry County zip codes, the IDPH zip-code level COVID-19 data were mapped onto the census block group data organized within the USEPA EJSCREEN Tool. Mapping and analysis of spatial relationships were performed using ESRI ArcGIS Pro 2.5.2 and statistical data analysis was performed using SAS's JMP 15 software (ArcGIS, 2020; SAS, 2019).

As of June 23, 2020, the total number for severe acute respiratory syndrome coronavirus 2 (SARS-CoV-2)/COVID-19 (tests conducted in Illinois was 1,379,003 and the total number of positive cases of COVID-19 was 137,224. The COVID-19 data reported by IDPH are aggregated by county and zip code (IDPH). For the 1,383 zip codes located within Illinois, IDPH provides COVID-19 data for the number of people tested and the number of positive cases for 563 zip codes (41%). The study area, which is comprised of Cook, DuPage, Lake, Will, Kane, and McHenry Counties, contains a total of 298 zip codes (53%); 7 of these zip codes (i.e., 60511, 60519, 60539, 60180, 60072, 60144, 60034) did not have any COVID-19 data provided by IDPH.

The COVID-19 zip code level incidence rate per 100,000 people was calculated by dividing the positive cases of COVID-19 in each study zip code reported by the IDPH by the population size reported by the US Census Bureau in its 2018 American Community Survey 5-Year Estimate for each zip code (USCB, 2018a, b).

The USEPA EJSCREEN is an EJ mapping and screening tool, which combines environmental and demographic indicators, to identify areas where people are most vulnerable or likely to be exposed to different types of pollution and to foster environmental justice analysis (USEPA). EJSCREEN contains 11 environmental indicators which include: USEPA's National Air Toxics Assessment (NATA) air toxics cancer risk; NATA respiratory hazard index; NATA diesel PM (DPM) concentration (μg/m^3^); particulate matter with aerodynamic size less than 2.5 μm (PM_2.5_) annual average concentration (μg/m^3^); ozone seasonal average concentration (μg/m^3^); traffic proximity and volume; lead paint represented by the percentage of houses built before 1960; proximity to potential accident Risk Management Plan (RMP) sites; proximity to hazardous waste treatment, storage, and disposal facilities (TSDFs); proximity to National Priority List (NPL) sites; and wastewater discharge toxicity. EJSCREEN also includes 6 demographic indicators which include percentage of the population below twice the federal poverty level (low-income); all people other than non-Hispanic white-alone individuals (minority); percentage of people age 25 or older without a high school diploma; percentage of people within a household in which all members age 14 and older speak English less than “very well” (linguistic isolation); percentage of people under the age of 5; and percentage of people over the age of 64 (USEPA, 2019a). The EJSCREEN data, which is organized by census block group, was downloaded from the USEPA website and data for the state of Illinois, including the study area, were extracted (USEPA, 2019b). There are a total of 5,824 and 9,689 census block groups in the six study counties and in the state of Illinois, respectively (USCB, 2018b).

In order to assess the COVID-19 zip code level incidence rate within the context of census block group EJSCREEN data, first the geographic scale of analysis must match. For this, the EJSCREEN indicators within each census block group were aggregated into zip codes via a spatial join. Specifically, if a centroid (geometric center) of a census block group fell within a zip code, the EJSCREEN demographic and environmental indicators from that census block group were assigned to that zip code. If the centroid of multiple census block groups fell within a zip code, the demographic population indicators were added, demographic percentages were recalculated, and the environmental indicator values were averaged across all census block groups within that zip code.

After all variables were aggregated into zip codes, the summary statistics and distributions of each of the eighteen variables (i.e., 11 environmental indicators, 6 demographic indicators, and COVID-19 incidence rates) across Cook, DuPage, Lake, Will, Kane, and McHenry counties were evaluated and ten quantile ranges were calculated for each variable. Values that fell within a specific quantile range were assigned a rank of 1–10 as shown in Table 1 (e.g., if the indicator value within a zip code value fell between the 10^th^ – 25^th^ percentile, it would receive a rank of 4).

**Table 1:** Quantiles and rankings for each variable/indicator.

Quantiles (%)	Assigned Rank Value	Aggregate Qualitative Rank
0 (Min)–0.5	1	LOW
0.5–2.5	2	
2.5–10	3	LOW – MEDIUM
10–25 (Q1)	4	
25 (Q1)–50 (Median)	5	MEDIUM
50 (Median)–75 (Q3)	6	
75–90	7	MEDIUM – HIGH
90–97.5	8	
97.5–99.5	9	HIGH
99.5–100 (Max)	10	

If the zip code value for an EJSCREEN indicator or COVID-19 incidence rate was 0, which occurred in five of the eighteen variables (i.e., wastewater discharge toxicity, percentage of people age 25 or older without a high school diploma; linguistic isolation, percentage of people under the age of 5, and COVID-19 incidence rate), they were assigned a rank of 0 for that indicator/rate.

The total ranks across all variables were assigned, as described above, and combined to form two primary assessment category groups (i.e., combined total 17 EJSCREEN environmental and deomographic indicator ranks; and COVID-19 incidence rate ranks) with varying possible minimum and maximum totals to explore the association between COVID-19 rates and ECSCREEN environmental and demographic variables (i.e., EJSCREEN indicator ranks: minimum 13, maximum 170; and combined total EJSCREEN indicator (environmental and demographic) ranks and COVID-19 incidence rate ranks: minimum 13, maximum 180).

For each category group defined above, an additional distribution analysis of the zip code ranks was conducted to determine the quantile breakdown of the rank totals. Based on the quantile results, zip codes were assigned to an aggregate qualitative rank of “Low”, “Low-Medium”, “Medium”, “Medium-High”, or “High”. The corresponding relationship between the distribution, ranks, and aggregate qualitative rank assignments is shown in Table 1.

**Figure 1:**
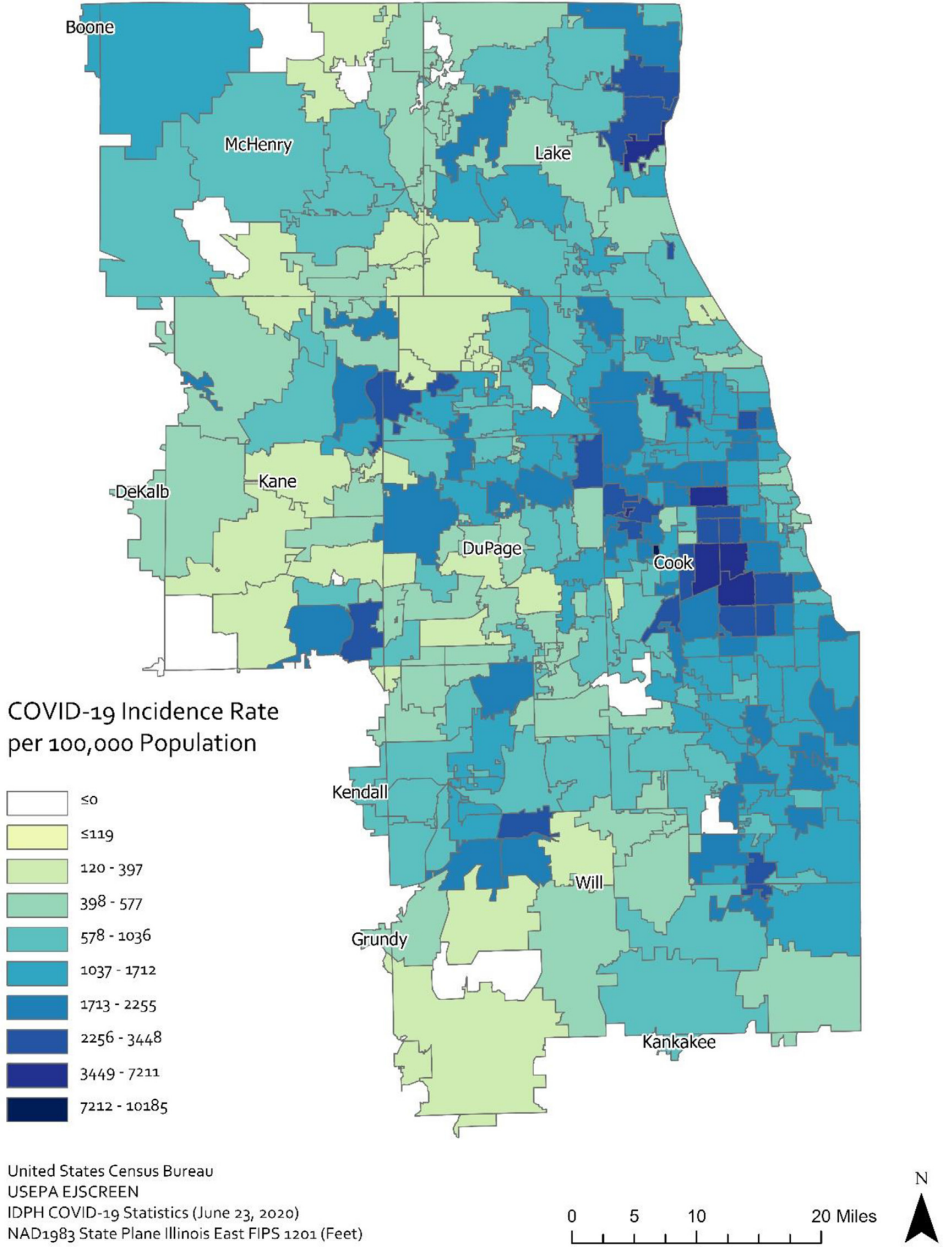
COVID-19 incidence rate in Cook, DuPage, will, Lake, Kane, and McHenry County Illinois.

**Figure 2:**
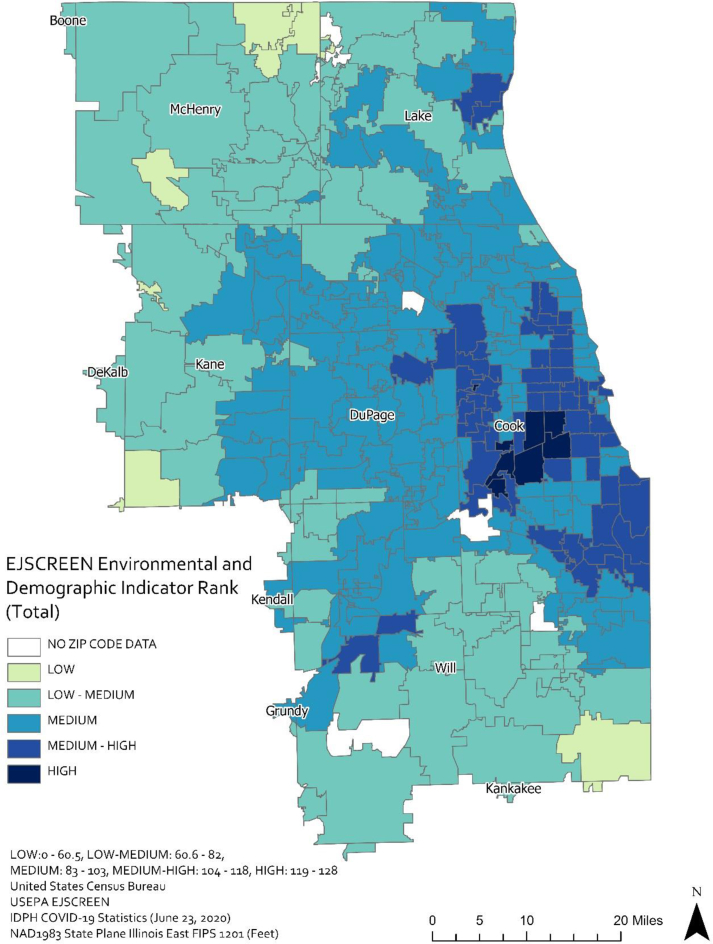
EJSCREEN environmental/demographic indicator ranking (Total) in Cook, DuPage, will, Lake, Kane, and McHenry County Illinois.

**Figure 3:**
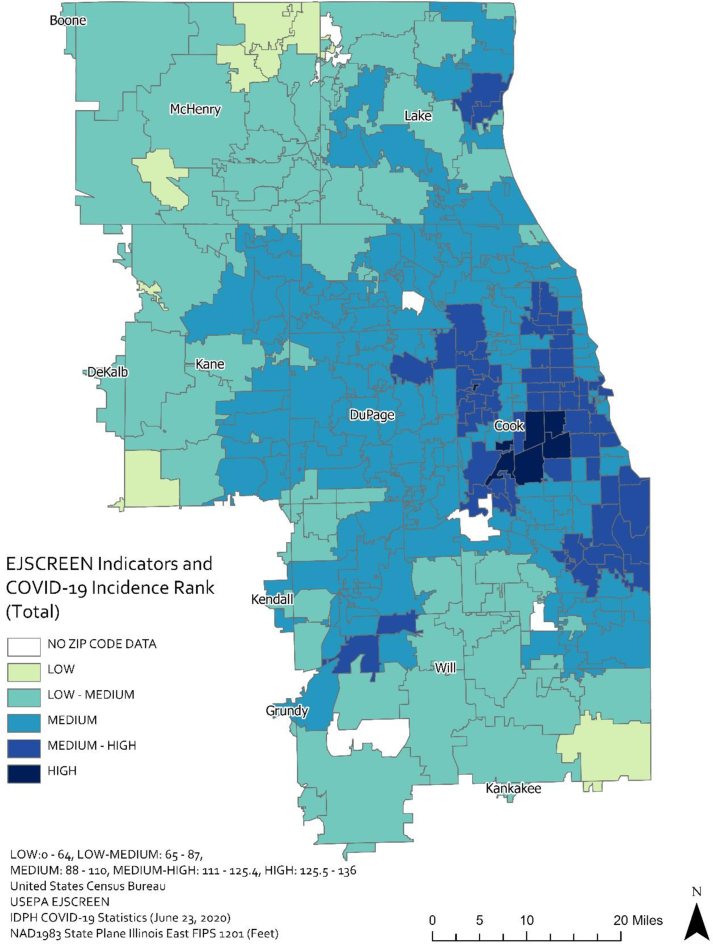
EJSCREEN environmental/demographic indicator and COVID-19 incidence ranking (Total) in Cook, DuPage, will, Lake, Kane, and McHenry County Illinois.

Rank results for the qualitative category groups shown in Table 1 were spatially examined at the zip code level for Cook, DuPage, Lake, Will, Kane, and McHenry Counties. While Figure 1 shows the spatial distribution of the zip code level COVID-19 incidence rate data, Figure 2 shows the spatial distribution of total environmental and demographic EJSCREEN indicator ranks. Figures 1 and 2 data were superimposed in Figure 3, which shows the combined total environmental and demographic EJSCREEN indicator ranks along with the COVID-19 incidence rate ranks spatially at the zip code level.

To determine if there is a relationship between calculated EJSCREEN indicator ranks and the COVID-19 incidence rate ranks, non-parametric Spearman's Rho (ρ) correlation coefficients were calculated for the COVID-19 incidence rate ranks vs. each specific EJSCREEN environmental and demographic indicator ranks; total environmental indicator ranks; total demographic indicator ranks; and combined environmental and demographic indicator ranks. The interpretation of the correlation coefficients was performed following analysis of Mukaka and Hinkle (Mukaka, 2012; Hinkle et al., 2002) (see Table 2).

**Table 2:** Correlation coefficients results.

Variable	By Variable	Spearman ρ Coefficient	Prob> |p|
**EJSCREEN Indicator Ranks and COVID-19 Incidence Rate Ranks**			
EJSCREEN Demographic Indicator Rank	COVID-19 Incidence Rate Rank	0.75	<.0001
EJSCREEN Environmental (total) Indicator Rank	COVID-19 Incidence Rate Rank	0.45	<.0001
EJSCREEN (total) Indicator Rank	COVID-19 Incidence Rate Rank	0.65	<.0001
**EJSCREEN Environmental Indicator Ranks and COVID-19 Incidence Rate Ranks**			
NATA Air Toxics Cancer Risk	COVID-19 Incidence Rate Rank	0.40	<.0001
NATA Respiratory Hazard Index	COVID-19 Incidence Rate Rank	0.44	<.0001
NATA Diesel PM (DPM)	COVID-19 Incidence Rate Rank	0.41	<.0001
Particulate Matter (PM_2.5_)	COVID-19 Incidence Rate Rank	0.39	<.0001
Ozone	COVID-19 Incidence Rate Rank	0.10	0.0976
Traffic Proximity and Volume	COVID-19 Incidence Rate Rank	0.35	<.0001
Lead Paint	COVID-19 Incidence Rate Rank	0.49	<.0001
Proximity to RMP Sites	COVID-19 Incidence Rate Rank	0.44	<.0001
Proximity to TSDFs	COVID-19 Incidence Rate Rank	0.41	<.0001
Proximity to NPL Sites	COVID-19 Incidence Rate Rank	-0.21	0.0004
Wastewater Discharge	COVID-19 Incidence Rate Rank	0.15	0.0079
**EJSCREEN Demographic Indicator Ranks and COVID-19 Incidence Rate Ranks**			
Low-Income	COVID-19 Incidence Rate Rank	0.70	<.0001
Minority	COVID-19 Incidence Rate Rank	0.71	<.0001
Less than high school education	COVID-19 Incidence Rate Rank	0.72	<.0001
Linguistic isolation	COVID-19 Incidence Rate Rank	0.48	<.0001
Individuals under age 5	COVID-19 Incidence Rate Rank	0.29	<.0001
Individuals over age 64	COVID-19 Incidence Rate Rank	-0.13	0.0297
**Race and COVID-19 Incidence Rate**			
Percent Not Hispanic or Latino: White alone	COVID-19 Incidence Rate	-0.76	<.0001
Percent Not Hispanic or Latino: Black or African American alone	COVID-19 Incidence Rate	0.52	<.0001
Percent Not Hispanic or Latino: American Indian and Alaska Native alone	COVID-19 Incidence Rate	0.16	0.0059
Percent Not Hispanic or Latino: Asian alone	COVID-19 Incidence Rate	-0.11	0.0512
Percent Not Hispanic or Latino: Native Hawaiian and Other Pacific Islander alone	COVID-19 Incidence Rate	-0.03	0.6629
Percent Not Hispanic or Latino: Some other race alone	COVID-19 Incidence Rate	0.16	0.0046
Percent Two or more races	COVID-19 Incidence Rate	-0.07	0.2369
Percent Hispanic or Latino	COVID-19 Incidence Rate	0.50	<.0001

An additional analysis was conducted to calculate the correlation coefficient between COVID-19 incidence rate and race for each zip code using the demographic data from the US Census that included the American Community Survey (ACS) 5-yr Estimates (2018) (USCB, 2018b). The racial groups analyzed included the total population estimate for Not Hispanic or Latino; White alone; Black or African American alone; American Indian and Alaska Native alone; Asian alone; Native Hawaiian and Other Pacific Islander alone; Some other race alone; and Two or more races and total Hispanic or Latino population. The county level percentages of each racial group estimated by the US Census (2019) and the US Census definition of each racial and ethnic variable are shown in Table 3. In this study, the percentage of each racial group within each zip code was calculated by dividing the population estimate of the racial group by the total zip code population.

**Table 3:** County race and Hispanic Origan characteristics, US census population estimates (V2019).

County	Population Estimates (2019)	White (Not Hispanic or Latino) (%) a	Black or African American (%)^b^	American Indian and Alaska Native (%) c	Asian (%) d	Native Hawaiian and Other Pacific Islander (%) e	Two or more races (%) f	Hispanic or Latino (%) g
Cook	5,150,233	42.0	23.8	0.7	7.9	0.1	2.0	25.6
DuPage	922,921	66.0	5.3	0.4	12.7	0.1	2.1	14.6
Lake	696,535	60.6	7.5	0.9	8.4	0.1	2.2	22.4
Will	690,743	62.5	12.2	0.5	6.0	0.1	2.0	18.2
Kane	532,403	56.6	6.0	1.0	4.4	0.1	2.0	32.4
McHenry	307,774	80.1	1.8	0.5	3.0	0.1	1.7	13.9

U.S. Census QuickFacts (V2019)

U.S. Census Bureau, Population Estimates Program (PEP). Updated annually.

a **“White**. A person having origins in any of the original peoples of Europe, the Middle East, or North Africa. It includes people who indicate their race as "White" or report entries such as Irish, German, Italian, Lebanese, Arab, Moroccan, or Caucasian.”

b **“Black or African American**. A person having origins in any of the Black racial groups of Africa. It includes people who indicate their race as "Black or African American," or report entries such as African American, Kenyan, Nigerian, or Haitian.”

c **“American Indian and Alaska Native**. A person having origins in any of the original peoples of North and South America (including Central America) and who maintains tribal affiliation or community attachment. This category includes people who indicate their race as "American Indian or Alaska Native" or report entries such as Navajo, Blackfeet, Inupiat, Yup'ik, or Central American Indian groups or South American Indian groups.”

d **“Asian**. A person having origins in any of the original peoples of the Far East, Southeast Asia, or the Indian subcontinent including, for example, Cambodia, China, India, Japan, Korea, Malaysia, Pakistan, the Philippine Islands, Thailand, and Vietnam. This includes people who reported detailed Asian responses such as: "Asian Indian," "Chinese," "Filipino," "Korean," "Japanese," "Vietnamese," and "Other Asian" or provide other detailed Asian responses.”

e **“Native Hawaiian and Other Pacific Islander**. A person having origins in any of the original peoples of Hawaii, Guam, Samoa, or other Pacific Islands. It includes people who reported their race as "Fijian," "Guamanian or Chamorro," "Marshallese," "Native Hawaiian," "Samoan," "Tongan," and "Other Pacific Islander" or provide other detailed Pacific Islander responses.”

f **“Two or more races**. People may choose to provide two or more races either by checking two or more race response check boxes, by providing multiple responses, or by some combination of check boxes and other responses. For data product purposes, "Two or More Races" refers to combinations of two or more of the following race categories: "White," "Black or African American," American Indian or Alaska Native," "Asian," Native Hawaiian or Other Pacific Islander," or "Some Other Race".

g **“Hispanics or Latino** refers to a person of Cuban, Mexican, Puerto Rican, South or Central American, or other Spanish culture or origin regardless of race. This includes people who reported detailed Hispanic or Latino groups”.

## 3 Results

The COVID 19 incidence rate as reported by the Illinois Department of Public Health is the incidence rate of the positive results for SARS-CoV-2 testing and will be referred to as COVID-19 incidence rate.

The zip code level COVID-19 incidence rate (Figure 1), combined total EJSCREEN environmental and demographic indicator ranks (Figure 2), and combined total EJSREEN environmental and demographic indicator ranks and COVID-19 incidence rate ranks (Figure 3) were geographically and spatially represented in Figures 1, 2, 3. All seven zip codes assigned a “high” qualitative rank for total EJSCREEN and COVID-19 incidence rate ranks (i.e., 60501, 60804, 60534, 60165, 60623, 60632, and 60638) were located within Cook County.

Many of these zip codes are current or former industrial corridors and include industrial sources such as corn refining plant, a roofing and asphalt plant, and a protective coating manufacturing facility (zip code 60501); a manufacturer of industrial carbon pitch, coal tar distillates and refined tars, wood.

Preservatives, phthalic anhydride, specialty chemicals and commercial grade rood products and a rail intermodal yard (zip code 60804); an industrial manufacturing and distribution center (zip code 60534); soap and detergent manufacturing plant (zip code 60165); a number of fabricated metals industries (zip code 60623); fabricated and primary metals industry and a manufacture of automative aftermarket fluids and additives (zip code 60632); and a number of fabricated and primary metal, chemical, petroleum, and packing industries (zip code 60638); per USEPA's Toxic Release Inventory (USEPA, 2021). In addition, demolition of a former coal-powered power plant resulted in an acute air pollution episode in the midst of the COVID-19 pandemic in April, 2020 in one of these neighborhoods, which disproportionately increase the pollution burden in this community (zip code 60623). In addition, based on the primary, secondary and tertiary race/ethnicity populations, percentage living in poverty, and median household income reported by the US Census ACS 2019 5-year estimates and listed in Table 4, they are also socio-economically disadvantaged neighborhoods (IL Demographics, 2019).

**Table 4:** Neighborhood characteristics of zip codes assigned a “high” qualitative rank for total EJSCREEN and COVID-19 incidence rate ranks.

Zip Code	Population	Race/Ethnicity			Living in Poverty (%)	Median Household Income
		Primary (%)	Secondary (%)	Tertiary (%)		
60501	11,874	72.5 Hispanic	16.2 White	7.1 Black	15.1	$53,258
60804	82.383	89.6 Hispanic	6.3 White	3.0 Black	13.4	$49,412
60534	10,452	51.8 Hispanic	41.6 White	4.6 Black	9.6	$60,601
60165	4,894	89.9 Hispanic	4.5 White	2.6 Black	13.1	$59,778
60623	81,283	66.8 Hispanic	29.7 Black	2.9 White	25.7	$32,460
60632	89,857	83.0 Hispanic	8.5 White	6.9 Asian	17.5	$44,924
60638	58,669	50.4 Hispanic,	44.3 White	3.7 Black	7.4	$68,089

Health indicator statistics across these seven zip codes including the three-year average rate per 10,000 of adult emergency department visits for asthma from 2016 to 2018 (60501: 48.36, 60804: 37.11, 60534: 46.9, 60165: 51.29, 60623: 102.22, 60632: 26.21, and 60638: 23.73) indicated that four zip code rates were above the state rate of 38.33 per 10,000 and the average COVID-19 rates as of June 23, 2020 were disproportionately higher (3,002/100,000 population) than the overall average COVID-19 rates across the study area (1,246/100,000 population), highlighting disparate risks for health indicators including asthma and COVID-19 spatially (IDPH, Public Health Community Map).

### 3.1 COVID-19 incidence rate and EJSCREEN indicator relationship

The relationship between EJSCREEN indicator ranks and the COVID-19 incidence rate ranks showed a high positive correlation between EJSCREEN demographic indicator ranks and COVID-19 incidence rate ranks (Spearman's ρ = 0.75) and moderate positive correlations between the total EJSCREEN environmental indicator ranks (Spearman's ρ = 0.45) and combined EJSCREEN environmental and demographic indicator ranks and COVID-19 incidence rate ranks (Spearman's ρ = 0.65).

Evaluation of the correlation between EJSCREEN environmental indicator ranks and COVID-19 incidence rate ranks found low positive correlations between NATA air toxics cancer risk (Spearman's ρ = 0.40), NATA respiratory hazard index (Spearman's ρ = 0.44), NATA DPM (Spearman's ρ = 0.41), PM_2.5_ (Spearman's ρ = 0.39), traffic proximity and volume (Spearman's ρ = 0.35), lead paint (Spearman's ρ = 0.49), proximity to RMP sites (Spearman's ρ = 0.44), and proximity to TSDFs (Spearman's ρ = 0.41) and COVID-19 incidence rate ranks. All other correlations were negligible (i.e., <0.30).

Evaluation of the correlation between EJSCREEN demographic indicator ranks and COVID-19 incidence rate ranks found high positive correlations between percentage of the population below twice the federal poverty level (Spearman's ρ = 0.70), all people other than non-Hispanic white-alone individuals (Spearman's ρ = 0.71), and percentage of people age 25 or older without a high school diploma (Spearman's ρ = 0.72) and COVID-19 incidence rate rank; and a low positive correlation between percentage of people within a household in which all members age 14 and older speak English less than “very well” and COVID-19 incidence rate rank. All other correlations were negligible (i.e., <0.30).

### 3.2 COVID-19 incidence rate and race relationship

Due to positive correlations between COVID-19 incidence rate ranks and EJSCREEN demographic indicator ranks (Spearman's ρ = 0.75), individual minority indicators (Spearman's ρ = 0.71), and individual linguistic isolation (Spearman's ρ = 0.48) indicators, an additional analysis was conducted to assess the correlation between COVID-19 incidence rate and race. Correlation coefficients between zip code level percent Black/African American (non-Hispanic or Latino) or Hispanic/Latino and COVID-19 incidence rate were moderate and positive (Spearman's ρ = 0.52 and 0.50). Furthermore, a high negative correlation was found between “Percent Not Hispanic or Latino: White alone” and COVID-19 incidence rate rank (Spearman's ρ = -0.76). All other correlations were negligible (i.e., <0.30).

## 4 Discussion and conclusions

The results of this analysis support the emerging scientific evidence demonstrating positive relationships between severe acute respiratory syndrome coronavirus 2 (SARS-CoV-2)/COVID-19 incidence rates and a number of environmental and demographic EJ indicators (USEPA, Environmental Justice). Additionally, the unequal burden of reported COVID-19 on disadvantaged communities has previously been reported.^29^ More specifically, a moderately positive correlation was found between COVID-19 incidence rate ranks and total EJSCREEN environmental and demographic EJ indicator ranks, which could be a marker of a community vulnerability or distress index. These communities also have a higher Social Vulnerability Index (SoVI) which takes into consideration social inequities that include lack of resources, information and knowledge; limited English language skills; limited access to political power; and beliefs and customs (Cutter et al., 2003).

Furthermore, the positive correlation between COVID-19 incidence rate ranks and individual linguistic isolation (Spearman's ρ = 0.48) may be linked to the lack of timely receival of COVID-19 health risk and transmission information by subpopulations who do not speak English. The California Office of Environmental Health and Hazard Assessment (2021) categorizes linguistic isolation as being adults with lack of proficiency in English may have difficulty in interacting with people who provide social services and medical care and may not hear or understand important information communicated to the public in an emergency.

Although there was a significant moderate association between COVID-19 incidence rate ranks and the EJSCREEN environmental indicator ranks, we observed a low positive but significant association between COVID-19 incidence rate ranks and modeled NATA (DPM and PM_2.5_ concentrations estimated based on EPA's National Emissions Inventory (NEI) data. This finding also provides supporting evidence to findings of previous published research that reported a positive association between COVID-19 incidence rates and ambient PM_2.5_ exposures and uniquely delineates DPM as another risk factor for COVID-19 (Zhu et al., 2020; Wu et al., 2020a, b, c; Zhang et al., 2021; Travaglio et al., 2021).

In addition, we observed a high negative correlation between “Percent Not Hispanic or Latino: White alone” and COVID-19 incidence rate (Spearman's ρ = -0.76) which could be potentially partly attributed to lower COVID-19 incidence rates in areas with a higher percentage of “Not Hispanic or Latino: White alone” population and/or a lack of statistical power due to small sample size in areas with higher COVID-19 incidence rates and predominantly “Not Hispanic or Latino: White alone” population zip codes. “Not Hispanic or Latino: White alone populations” is defined by the US Census as “individuals who responded "No, not Spanish/Hispanic/Latino" and who reported "White" as their only entry in the race question”. “White” is defined by the US Census as “A person having origins in any of the original peoples of Europe, the Middle East, or North Africa. It includes people who indicate their race as "White" or report entries such as Irish, German, Italian, Lebanese, Arab, Moroccan, or Caucasian.” (U.S. Census Bureau, Population Estimates Program (PEP). Updated annually. 2021).

Besides air pollution, a number of studies have also identified demographic variables associated with COVID-19 cases (Madrazo Cabo et al., 2020). The importance of air quality and socioeconomic factors must be further understood for more targeted public health responses to communities that are disproportionately affected by high rates of positive SARS-CoV2.

A limitation of our research is correlation of comorbidities for other chronic medical conditions of individuals, such as hypertension and diabetes, who test positive for SARS-CoV-2 which lead to COVID 19. Additionally, another limitation is the underestimation of positive individuals who never get tested because of being asymptomatic or having mild symptoms or limited access to health care or testing sites. Utilizing a probability analysis, researchers found that there is an underestimate of actual individuals infected with severe acute respiratory syndrome coronavirus 2 (SARS-CoV-2) which may be up to 9 times the confirmed number of those tested (Wu et al., 2020a, b, c). Accordingly, we call for future research that aims to uncover role of air pollution in COVID-19 incidence and mortality in general and in COVID-19 disparities observed for socio-economically disadvantaged populations for other geographic locations, as documented hire for Northern Illinois counties. Further research remains to be performed to accurately confirm the number of individuals who remain asymptomatic or do not get tested.

## Declarations

### Author contribution statement

Martha Menchaca, Frank Pagone, Serap Erdal: Conceived and designed the experiments; Analyzed and interpreted the data; Contributed reagents, materials, analysis tools or data; Wrote the paper.

### Funding statement

This research did not receive any specific grant from funding agencies in the public, commercial, or not-for-profit sectors.

### Data availability statement

Data associated with this study has been deposited at https://www.dph.illinois.gov/covid19/covid19-statistics and https://www.epa.gov/ejscreen/overview-environmental-indicators-ejscreen.

### Declaration of interests statement

The authors declare no conflict of interest.

### Additional information

No additional information is available for this paper.
